# *Pseudomonas aeruginosa* Virulence Bacteriophage Isolated From Inflammatory Mouse Feces Exhibits Bactericidal Activity in Infected Wounds of a Mouse Model

**DOI:** 10.1155/ijm/4451708

**Published:** 2026-07-03

**Authors:** Uthaibhorn Singkham-In, Supadtra Pedcharat, Wilasinee Saisorn, Vorthon Sawaswong, Pornpimol phuengmaung, Asada Leelahavanichkul

**Affiliations:** ^1^ Department of Clinical Microbiology, Faculty of Medical Technology, Rangsit University, Pathum Thani, Thailand, rsu.ac.th; ^2^ Center of Excellence in Translational Research in Inflammatory and Immunology (CETRII), Chulalongkorn University, Bangkok, Thailand, chula.ac.th; ^3^ Interdisciplinary Program of Medical Microbiology, Graduate School, Chulalongkorn University, Bangkok, Thailand, chula.ac.th; ^4^ Interdisciplinary Program of Biomedical Sciences, Graduate School, Chulalongkorn University, Bangkok, Thailand, chula.ac.th; ^5^ Department of Biochemistry, Faculty of Science, Mahidol University, Bangkok, Thailand, mahidol.ac.th; ^6^ Department of Microbiology, Faculty of Medicine, Chulalongkorn University, Bangkok, Thailand, chula.ac.th; ^7^ Department of Medicine, Faculty of Medicine, Chulalongkorn University, Bangkok, Thailand, chula.ac.th

**Keywords:** bacteriophage, bacteriophage therapy, cecal ligation and puncture (CLP) model, *Pseudomonas aeruginosa*, *Pseudomonas aeruginosa*-infected wound model, wound infection

## Abstract

**Background:**

Recently, bacteriophages have risen as a potent therapy for superbug infections. The mammal gut demonstrates an interesting source of virulence bacteriophages. The gut with inflammation is phage‐rich; therefore, we primarily aimed to prove the concept that an inflammatory gut is a possible source of effective phages and to evaluate the efficacy of the candidate phage against *Pseudomonas aeruginosa* in vitro and in a mouse model of infected wounds.

**Results:**

The gut microbiome of cecal ligation and puncture (CLP) sepsis mice, an animal model of inflammation, showed a dominant presence of Podoviruses. CLP bacteriophages (CLP *Φ*1–*Φ*4), of which the CLP *Φ*4 possessed the broadest bactericidal activity (viable bacterial cell reduction in time‐kill study) against *P. aeruginosa* isolates. The CLP *Φ*4 specifically killed the *Pseudomonas aeruginosa* clinical (PACL) strain with two huge burst events. Although the CLP *Φ*4 had no effect on ex vivo mouse bone marrow‐derived macrophage (BMDM) cytokine gene expression and cytokine production, the CLP *Φ*4 attenuated the severity of the *P. aeruginosa*–infected wound mouse model after treatment. *P. aeruginosa* PACL exhibited significantly pathogenic characteristics in a mouse model, including excessive bacterial loads (in wounds and internal organs, indicating the systemic infection due to localized infected wound with *P. aeruginosa*), increased IL‐6 cytokine (in serum), upregulated IL‐6 expression (in wounds), and immune cell infiltration (in wounds), indicating severe inflammation. In the CLP *Φ*4 treatment alone, the wound tissues upregulated *IL-10* expression and recruited inflammatory cells. Interestingly, the three‐day CLP *Φ*4 treatment was adequate to eradicate *P. aeruginosa* PACL in the wounds and other internal organs. After treatment, the mouse serum cytokine showed a remarkably decreased IL‐6. Likewise, IL‐6 downregulation and IL‐10 upregulation were demonstrated in the treated wounds, suggesting an anti‐inflammatory shift. These results demonstrated the effectiveness (bacterial wound and internal organ clearance and cytokine modulation) of the CLP *Φ*4 in the *P. aeruginosa*–infected wound and systemic infection. Finally, the CLP *Φ*4 isolation verified a proof of concept that the irritated gut acts as a source of bacteriophages.

**Conclusions:**

The gut virome was a promising and interesting source of antimicrobial and immunomodulating bacteriophage.

## 1. Background

*Pseudomonas aeruginosa* has been remarkably a nosocomial pathogen in healthcare settings for decades [1]. Because of genetic diversity and metabolic adaptation, *P. aeruginosa* is a versatile organism that survives in extreme environments (chemical and desiccated surfaces), leading to hospital‐acquired infections, including bacteremia, pneumonia, skin, soft tissue, and wound infections [2]. The critical virulence that enables *P. aeruginosa* to emerge as a bug is biofilm formation [3]. *P. aeruginosa* forms biofilms on central venous catheters and endotracheal tubes, leading to catheter‐related bacteremia and pneumonia, respectively [4]. Moreover, *P. aeruginosa* establishes biofilms in the lung tissue of cystic fibrosis patients [5] and the wound tissue of chronic wound infections [6], indicating the plasticity of *P. aeruginosa* lifestyle within the human host. Apart from variable virulence, *P. aeruginosa* is responsible for multiple antimicrobial resistance, emerging as a difficult‐to‐treat resistant (DTR) organism. The DTR‐*P. aeruginosa* is resistant to numerous antimicrobial agents, especially the broadest beta‐lactams, carbapenems [7], shedding light on other novel therapies, including bacteriophages.

Bacteriophages are viruses that infect bacterial host cells and reproduce via either lytic or lysogenic cycles [8]. The lytic bacteriophages replicate within the hosts, leading to bacterial bursts. In contrast, lysogenic viruses integrate their genome into the host chromosomes, allowing for silent lifecycles within the host upon induction, during which viral genomes are excised and replicated to produce viral particles [8]. Therefore, lysogenic phages have fallen out of favor for bacteriophage therapy due to the potential for integration to cause undesirable gene transduction, such as the transfer of antimicrobial resistance or virulence genes [8, 9]. The natural resource of lytic bacteriophages typically contains a tremendous number of bacteria, especially in sewage, suggesting that these bacteriophages have a limited host range, primarily targeting environmental host strains rather than pathogenic bacteria [10]. Shift the focus to the living‐organism microbiome as an interesting source that restrains enormous bacteriophage specific to clinically isolated pathogenic bacteria [11]. Our previous study demonstrates that a healthy mouse gut microbiome is a promising novel source in which lytic bacteriophages isolated from feces efficiently eradicate *P. aeruginosa* in a pneumonia mouse model [12]. The *P. aeruginosa* bacteriophage isolated from feces exhibits a similar potential in intratracheal and intravenous therapeutic routes, indicating the flexible impact of viability and effectiveness. In addition, bacteriophage promotes anti‐inflammation by reducing cytokine release and the formation of neutrophil extracellular traps (NETs) [12]. Take the next step of mammal microbiota‐sourced bacteriophage.

The gut microbiome, a complicated community of microorganisms, influences host–microbe interaction and modulates homeostasis. The majority of microbiota, virome, of which approximately 97% are bacteriophages [13]. Bacteriophages play a direct role in shaping microbial diversity and composition [11]. The evolution of bacteria and bacteriophages is responsible for either antagonism or mutualism. Lytic bacteriophages and prophages can kill specific bacteria through the lytic cycle and induction, respectively, thereby decreasing the specified bacteria. On the other hand, some prophages and filamentous phages promote host benefits and survival, thereby increasing the specific bacteria. Controversially, the debate on the role of bacteriophages in the mammalian host immune response is on [13]. Generally, bacteriophages rarely penetrate the mucosal epithelium into the lumen and activate an immune response via dendritic cells [13]. However, the orally high dose of bacteriophages can induce phage‐specific antibody production, indicating an immune response to bacteriophages [14]. Mammalian immunity responds to bacteriophages in either an anti‐inflammatory response or an inflammatory response [13]. These data indicate that the approach to selecting applicable bacteriophages is a pathogen‐killing activity with appropriate immune induction.

Our previous study demonstrates that gut microbiome dysbiosis occurs during sepsis in the mouse model of cecal ligation and puncture (CLP), in which virome, particularly podovirus (a short and noncontractile‐tailed bacteriophage), predominates in the gut, suggesting an interesting and possible source for bacteriophage isolation [15]. Our primary objective is to prove the concept of an effective phage isolated from the inflammatory gut microbiome, and the secondary objective is to evaluate phage activity in vitro and in vivo study. Therefore, we (1) studied the gut microbiome of the CLP model, (2) isolated an effective bacteriophage specific to *P. aeruginosa* from the CLP mouse feces, (3) characterized in vitro killing activity and immune response of the CLP bacteriophage, and finally, (4) evaluated the in vivo activity of the CLP bacteriophage against a *P. aeruginosa*–infected mouse wound model.

## 2. Methods

### 2.1. Animal CLP Model, Fecal Virota, and Mouse Sample Analysis

The animal study protocol (2591001) was approved by the Institutional Animal Care and Use Committee of the Faculty of Medicine, Chulalongkorn University, following the US National Institutes of Health (NIH) guidelines for animal care and use. The mice (male 8‐week‐old C57BL/6) were purchased from Siam Nomura (Samut Sakhon, Thailand), allowing free access to water and chow before and after surgery. Then, CLP surgery (*n* = 4) was performed using the ligation at 12 mm from the cecal tip before puncturing twice with a 21‐gauge needle according to previous publications [16, 17]. In sham control mice (*n* = 3), the cecum was only identified through abdominal incision before suturing layer by layer with 6‐0 nylon sutures under isoflurane anesthesia. After surgery, 1 mL of prewarmed normal saline solution (NSS) with tramadol at 25 mg/kg/dose was subcutaneously administered at 6 and 18 h postsurgery. All mice were sacrificed at 24 h postsurgery using cervical dislocation under isoflurane anesthesia before postmortem sample collection, including feces in the descending colon and blood from cardiac puncture. The fecal virota analysis was performed according to a previous publication [15]. Briefly, fecal sample (50 mg) in 1 mL of phosphate‐buffered saline (PBS) was centrifuged, filtered through a 0.45‐*μ*m syringe filter (Sartorius, Göttingen, Germany), treated with the nuclease cocktail (Promega, Madison, Wisconsin, United States) before extraction of viral nucleic acid using the MagMAX Viral RNA Isolation kit (Applied Biosystems, Thermo Fisher Scientific, Waltham, Massachusetts, United States). The cDNA was constructed based on the RevertAid First Strand cDNA Synthesis Kit (Thermo Fisher Scientific) with 100 pmol of Sol A random primer: 5 ^′^‐GTT TCCCAC TGG AGG ATA NNN NNN NNN‐3 ^′^ following the manufacturer′s protocol. The random amplification of cDNA was performed based on a polymerase‐chain reaction (PCR) comprising 2 *μ*M of Sol B primer:5 ^′^‐GTT TCC CAC TGG AGG ATA‐3 ^′^. The random amplified products were used for DNA library preparation using NEBNext Ultra II DNA Library Prep Kit for Illumina (New England Biolabs, United States) that was paired‐end sequenced (2 × 250 cycles) using the Illumina MiSeq sequencing platform with 10% PhiX spike‐in. The raw FASTQ reads were quality‐checked by FastQC, trimmed by Trimmomatic (Version 0.36), and mapped the reads against the reference mouse genome (GRCm38) using Bowtie2. The unmapped reads were de novo assembled by EnsembleAssembler. The contigs were BLASTx search against the viral protein database (collected from ftp://ftp.ncbi.nih.gov/refseq/release/viral/) using with e−10 E − value cutoff. The taxonomically classified contigs were subsequently used as the reference sequence for mapping the viral reads to count the hits of each viral taxon. The datasets of virome analysis presented in this study can be found in online repositories. The name of the repository and accession number can be found below: NCBI Sequence Read Archive; PRJNA838435 https://www.ncbi.nlm.nih.gov/bioproject/PRJNA838435). For the quantification of fecal viruses, the filtrated samples were diluted and stained with SYBR Gold for DNA viruses and SYBR Green II for RNA viruses (Thermo Fisher Scientific). For the serum sample, creatinine and alanine transaminase were measured by QuantiChrom (DICT‐500) and EnzyChrom Alanine Transaminase assay (EALT‐100) (BioAssay, Hayward, California, United States), whereas serum cytokines (TNF‐*α* and IL‐6) were analyzed by enzyme‐linked immunosorbent assays (ELISA) (Invitrogen, Carlsbad, California, United States).

### 2.2. Bacterial Strains, Growth Conditions, Imipenem Susceptibility Testing, and Genetic Relatedness Study

*P. aeruginosa* clinically isolated (*n* = 40) from the patients admitted in the King Chulalongkorn Memorial Hospital of the Department of Microbiology, Faculty of Medicine, Chulalongkorn University, under the institutional review board (IRB) number MDCU‐IBC012/2025 from the Faculty of Medicine, Chulalongkorn University, according to the Declaration of Helsinki, were included in this study. All data (patient data and source of the organisms) were anonymized with the waiver of the requirement for informed consent by the ethics committee. Additionally, *P*. *aeruginosa* ATCC 27853 and PAO1, reference strains, were also used. All bacterial strains frozen in glycerol stock at −80°C were grown in trypticase soy broth (TSB; Difco, Becton, New Jersey, United States) and incubated at 37°C, 200 rpm for 18 h. Imipenem (Apollo Scientific, Bredbury, United Kingdom) susceptibility testing against all *P. aeruginosa* isolates was determined using the broth microdilution method with cation‐adjusted Mueller–Hinton broth (CAMHB) (Becton Dickenson BBL, New Jersey, United States). The susceptibility was interpreted according to the CLSI guideline 2025 [18] using *P. aeruginosa* ATCC 27853 as a reference control strain.

The genetic relatedness of *P. aeruginosa* clinical isolates was performed using the randomly amplified polymerase DNA (RAPD). Briefly, *P. aeruginosa* genomic DNA was extracted using the Tianamp DNA Kit (TIANGEN, Beijing, China) and amplified by PCR using primers listed in the Table S1 [19, 20]. The RAPD profiles were detected using agarose gel electrophoresis. The genetic relationship of *P. aeruginosa* strains was analyzed using the unweighted pair group method with the arithmetic mean (UPGMA) algorithm in the MEGA 11 program. *P. aeruginosa* belonging to the same clade was defined as > 90% similarity of RAPD profiles.

### 2.3. Bacteriophage Isolation, Host Range Determination, and Morphology Study

Bacteriophages isolated from CLP mice feces were isolated from CLP mice feces following a previous publication [12]. In brief, mouse feces were cocultured with *P. aeruginosa* (PA1, PA2, PA4, PA5, PA6, PA7, PA8, PA9, PA10, PA11, PA12, PA13, and PA14) mixtures as hosts of bacteriophages and incubated at 37°C for 18 h. The supernatants of the coculture mixture were collected after centrifugation and filtered through a 0.22 *μ*m filter. The presence of bacteriophages in the supernatant was confirmed using a plaque assay. The mixture of the supernatant (containing bacteriophages) and *P. aeruginosa* overnight culture (as bacteriophage host) was added to soft agar (0.7% TSB). Then, the mixture was poured onto a tryptic soy agar (TSA) plate and incubated at 37°C for 16 h to allow plaques (bacteriophages) to form. The isolated plaque was transferred into 1X SM buffer, and the bacteriophage was propagated as described above. To purify the bacteriophages, the cesium chloride density gradient method was performed as previously described [12].

The host range of bacteriophages was determined using a double‐layer agar method. Briefly, a mixture of individual *P. aeruginosa* (40 strains shown in Table 1) with soft agar was solidified on TSA plates. The purified bacteriophages were spotted on the double‐layer TSA agar plates of an individual *P. aeruginosa*. After incubation, plaque formation was observed, indicating the susceptible *P. aeruginosa* host.

**Table 1:** *P. aeruginosa* host range of CLP bacteriophages (*Φ*1–*Φ*4).

*P. aeruginosa* host strain	CLP *Φ*1	CLP *Φ*2	CLP *Φ*3	CLP *Φ*4	*P. aeruginosa* host strain	CLP *Φ*1	CLP *Φ*2	CLP *Φ*3	CLP *Φ*4
ATCC	**+**	**+**	**+**	**+**	**NP39**	**+**	**+**	**+**	**+**
PAO1	**−**	**−**	**−**	**−**	**NP46**	**+**	**+**	**+**	**+**
PACL	**+**	**+**	**+**	**+**	**NP51**	**−**	**−**	**−**	+
PA1	**+**	**+**	**+**	**+**	**NP52**	**−**	**−**	**−**	+
PA2	**+**	**+**	**+**	**+**	**NP53**	**+**	**+**	**+**	**+**
PA4	**+**	**+**	**+**	**+**	**NP54**	**+**	**+**	**+**	**+**
PA5	**+**	**+**	**+**	**+**	**NP55**	**+**	**+**	**+**	**+**
PA6	**+**	**+**	**+**	**+**	**NP56**	**+**	**+**	**+**	**+**
PA7	**+**	**+**	**+**	**+**	**NP57**	**+**	**+**	**+**	**+**
PA9	**−**	**−**	**+**	+	**NP60**	**+**	**+**	**+**	**+**
PA10	**−**	**−**	**−**	**−**	**NP62**	−	−	−	−
PA11	**−**	**−**	**+**	+	**NP67**	**−**	**−**	**−**	+
PA12	**−**	**−**	**−**	**−**	**NP82**	−	−	−	−
PA13	**−**	**−**	**+**	+	**NP85**	−	−	−	−
PA14	**−**	**−**	**+**	+	**NP95**	**+**	**+**	**+**	**+**
NP8	**−**	**−**	**+**	**+**	**NP98**	−	−	−	−
NP10	**−**	**−**	**−**	**−**	**NP100**	−	−	−	−
NP18	**+**	**+**	**+**	**+**	**NP101**	−	−	−	−
NP23	**+**	**+**	**+**	**+**	**NP102**	−	−	−	−
NP30	**+**	**+**	**+**	**+**	**NP107**	**+**	**+**	**+**	**+**

*Note:* The symbols (+) denote positive of plaque formation and (−) negative of plaque formation.

The morphology of bacteriophage, which was applied to a carbon film 200 mesh copper grids (Electron Microscopy Sciences, Pennsylvania, United States), negatively stained with 2% uranyl acetate, was observed under a transmission electron microscope (TEM) (JEM 1400 Plus, JOEL, Massachusetts, United States) at an accelerating voltage of 80 kV.

### 2.4. One‐Step Growth Curve and Bactericidal Activity Study

The one‐step growth curve of bacteriophage (CLP *Φ*4) was performed against *P. aeruginosa* PACL as previously described [12]. Briefly, *P. aeruginosa* PACL with CLP *Φ*4 at a multiplicity of infection (MOI) of 0.01 was cocultured in TSB at 37°C with shaking at 200 rpm for 10 min to adsorb. Then, the coculture was centrifuged, and the supernatant was removed. The bacterial cell pellet adsorbed with CLP *Φ*4 was resuspended with TSB and incubated at 37°C with shaking at 200 rpm for 150 min. Every 10 min during incubation, the mixture was sampled for evaluation of CLP *Φ*4 number using a plaque assay on a double‐layer agar. The latent period of CLP *Φ*4 was determined using the one‐step growth curve. The burst size of CLP *Φ*4 was calculated by the maximum number of CLP *Φ*4 after the burst divided by the initial number of CLP *Φ*4 (at the latent period) [21].

The bactericidal activity of CLP *Φ*4 against *P. aeruginosa* PACL was performed using a time‐kill study. Shortly, *P. aeruginosa* PACL (10^6^ CFU/mL) was cocultured with CLP *Φ*4 at the MOI of 0.01, 0.1, 1, 10, and 100 at 37°C with shaking at 200 rpm for 24 h. Every 2 h of incubation, the coculture mixture was sampled for 8 h and at 24 h of incubation to quantify the bacterial viable cells on TSA plates.

### 2.5. Bone Marrow‐Derived Macrophages (BMDMs) Cytokine Assay

BMDMs were prepared from bone marrow cells isolated from femurs of 8‐week‐old wild‐type C57BL/6 mice as previously described [22]. Briefly, the isolated marrow cells were cultured in Dulbecco′s Modified Eagle′s medium (DMEM; Gibco, Massachusetts, United States) supplemented with macrophage colony‐stimulating factor (M‐CSF) in a humidified 5% CO_2_ incubator at 37°C for 7 days. The BMDMs (10^6^ cells) seeded into each well of a 12‐well plate were cocultured with *P. aeruginosa* PACL alone (5 × 10^6^ CFU) (*n* = 6), CLP *Φ*4 (5 × 10^8^ PFU) (*n* = 6), the combination of *P. aeruginosa* PACL and CLP *Φ*4 (*n* = 6), or DMEM control (*n* = 6) in a humidified 5% CO_2_ incubator at 37°C for an hour. The medium supernatant was collected and evaluated for bacterial and bacteriophage quantification. After removing the media, the BMDMs were washed with fresh DMEM and incubated at 37°C for 6 h. The supernatant media collected for cytokine assay (TNF‐*α*, IL‐6, and IL‐10) were analyzed using ELISA assays (Invitrogen). The BMDMs were collected for cytokine gene expression (*iNOS* and *Arg-1*) by RT‐qPCR using Trizol reagent for RNA extraction, the High‐Capacity cDNA Reverse Transcription kit (Applied Biosystems) for cDNA synthesis, and the SYBR Green PCR Master Mix (Applied Biosystems) for quantification of the number of transcripts using primers listed in Table S1 [23, 24]. The relative number of transcripts was normalized with *β-actin* and calculated using the 2^−*ΔΔ*ct^ method.

### 2.6. Animal *P. aeruginosa*–Infected Wound Model and Mouse Sample Analysis

The treatment of *P. aeruginosa*–infected wound mouse model using bacteriophages protocol (2591001) was approved by the Institutional Animal Care and Use Committee of the Faculty of Medicine, Chulalongkorn University, following the US NIH guidelines, using a specific pathogen‐free mouse facility. The 8‐week‐old male C57BL/6 mice (Nomura Siam, Thailand) were used with free access to water and food for a *P. aeruginosa*–infected wound model as previously described [25]. Before wound induction, the mice orally received tramadol and freely accessed drinking water supplemented with tramadol during the experiment.

To construct the infected wound model, shaving the skin at the back of mice, disinfection with 10% povidone‐iodine, skin puncture (8 mm in diameter), and inoculation of *P. aeruginosa* (10^6^ CFU) were done under the isoflurane anesthesia. In the treatment protocol, the mice were randomly grouped into four conditions, including (1) sterilized control (neither *P. aeruginosa* PACL, nor CLP *Φ*4) (*n* = 5), (2) *P. aeruginosa* infection (*n* = 5), (3) CLP *Φ*4 alone (10^8^ PFU) (*n* = 5), and (4) treatment (*P. aeruginosa* infection (10^6^ CFU) with CLP *Φ*4 treatment (10^8^ PFU)) (*n* = 6). After wound induction for 2 h, CLP *Φ*4 was directedly applied to the wound of CLP *Φ*4 alone and the treatment groups. The wounds were covered with a daily change of 3 M Tegaderm films (1622 W) (3 M Science, United States) until sacrifice. Wound fluids were daily collected for bacterial and bacteriophage enumeration. The wound covered films were daily change as standard wound care protocol. On the third day, the mice were sacrificed using cervical dislocation under isoflurane anesthesia before postmortem sample collection, including spleen, kidney, liver, wound tissue, and blood from cardiac puncture.

Wound fluids and other organ samples collected for *P. aeruginosa* PACL count were diluted with sterile NSS and plated onto TSA for counting the viable cells. Moreover, the diluted wound fluids and other organs were spotted onto TSA double‐layer agar covering *P. aeruginosa* PACL to enumerate bacteriophage CLP *Φ*4. Mouse serum collected for cytokine assay (TNF‐*α*, IL‐6, and IL‐10) was analyzed using ELISA assays (Invitrogen). Wound tissues and other organs were extracted for total RNA, cDNA synthesis, and quantification of cytokine gene expression using RT‐qPCR as described above. Additionally, wound tissues preserved in 10% formalin were processed, cut into sections, and placed on a glass slide, followed by staining with hematoxylin and eosin. The histopathological analysis was evaluated based on wound diameter, infiltrating cells (cell moving into wound tissue), redness, and moisture with a score (0–3) [26]. The detailed scoring criteria are presented in Table S2, where higher scores indicate greater severity. The individual scores for each parameter were then summed to generate an overall wound severity score. A minimum total score (0) represented the least severe wound condition, and a maximum score [12] indicated the most severe condition.

### 2.7. Statistical Analysis

Statistical analysis was performed using the Statistical Package for Social Sciences software (SPSS 22.0, SPSS Inc., Illinois, United States) and GraphPad Prism Version 8.0 software (La Jolla, California, United States). Normality was assessed using the Shapiro–Wilk test. All data (nonnormally distributed) were presented as median with range. Differences among two or more than two groups were analyzed using the Mann–Whitney or the Kruskal–Wallis test, followed by Dunn′s multiple comparison, respectively. A *p* value < 0.05 was considered statistically significant.

## 3. Results

### 3.1. Virome Dynamic Change With Podoviridae Predominace in the CLP Gut

**Figure 1:**
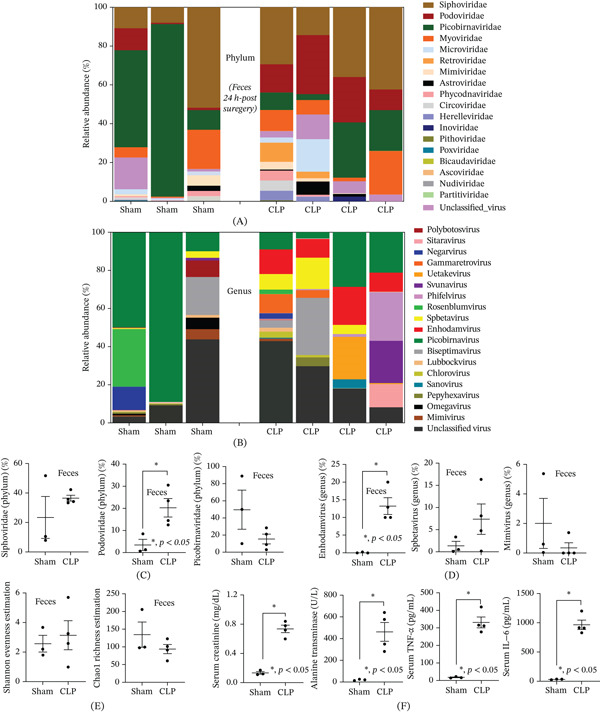
(A, B) The fecal abundance of the random sequence of viruses (virota or virome) from feces of mice after 24 h of sham (*n* = 3) or cecal ligation and puncture (CLP) (*n* = 4) surgery in phylum and genus levels together with the (C, D) graph presentation of some viruses in the phylum and genus levels, (E) alpha diversity (Shannon and Chao‐1 analysis), and (F) serum parameters (serum creatinine, alanine transaminase, TNF‐*α*, and IL‐6) are also indicated (*n* = 3 and 4 for sham and CLP, respectively).

To see if the microenvironment in the host alters the abundance of some viruses in the gut, a CLP sepsis model was developed before the determination of fecal virota and blood collection. Virome analysis in the phylum and genus from CLP mice after 24‐h surgery revealed a comparable population of viruses in the feces (Figure 1A,B). However, the feces revealed a prominent difference in virus belonging to Podoviridae (phylum) and *Enhodamvirus* (genus) between the sham control and the sepsis CLP mice (Figure 1C,D). According to the alpha diversity study using Shannon analysis, virota of the sham and CLP mice displayed a relative abundance of viruses (Figure 1E). Comparable to the Chao‐1 analysis, indicating the viral species richness in the sham relative to that of sepsis mouse feces (Figure 1E). These results suggested the specific change (Podoviridae predominant) in the bacteriophage community composition, potentially driven by inflammation (sepsis), while maintaining overall community richness and evenness.

In the sepsis severity study, mouse serum creatinine significantly surged in sepsis CLP mice, indicating kidney injury (Figure 1F). Moreover, alanine transaminase in CLP mouse serum was notably raised, denoting liver injury (Figure 1F). Concordantly, proinflammatory cytokines, including TNF‐*α* and IL‐6, in the serum of the CLP mice were strongly rising, supporting the inflammation (Figure 1F). Therefore, the virome study and sepsis parameters demonstrated the association between Podoviridae dominant in the CLP gut and severe sepsis inflammation.

### 3.2. Isolation of Bacteriophages From CLP Mouse Feces With a Candidate Phage, CLP *Φ*4

**Figure 2:**
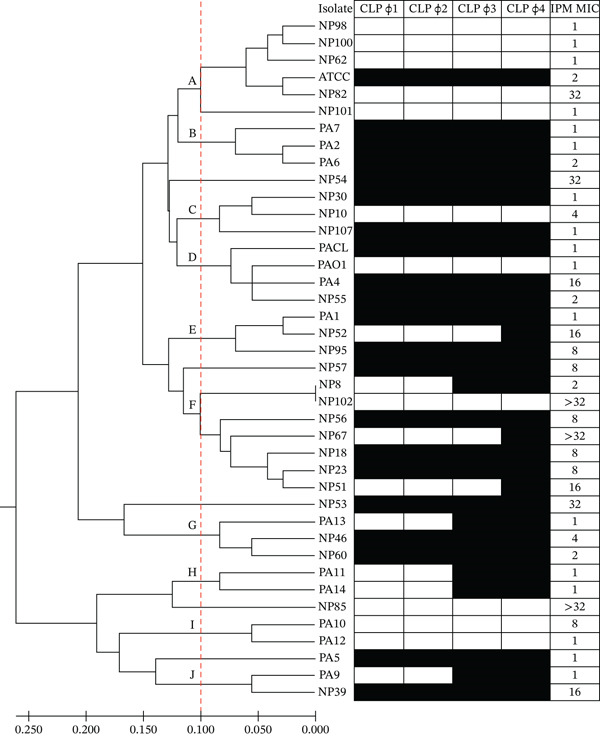
Genetic relatedness and imipenem susceptibility of *P. aeruginosa* strains (*n* = 40) for bacteriophage host range study. A dendrogram of genetic similarity based on RAPD profiles was generated by the MEGA 11 program using the UPGMA algorithm. A red‐dashed line indicated > 90% similar RAPD pattern.

As such, four different bacteriophages (CLP *Φ*1, CLP *Φ*2, CLP *Φ*3, and CLP *Φ*4) isolated from CLP mouse feces were evaluated for their host range using 40 *P. aeruginosa* strains (Table 1). The CLP *Φ*4 displayed the broadest host range against *P. aeruginosa* (72.5%) compared with CLP *Φ*3 (65.0%). Although the CLP *Φ*1 and *Φ*2 showed the narrowest host range (52.5%). To confirm the broad host range of the bacteriophages, the genetic relatedness of host strains, *P. aeruginosa*, was characterized using RAPD (Figures S1, S2, and S3). Among 40 isolates, there were 10 clades (clades A–J) and six singletons (*P. aeruginosa* NP101, NP54, NP57, NP53, NP85, and PA5) (Figure 2). The CLP *Φ*4 had an expanded activity against *P. aeruginosa* belonging to clades E (NP52) and F (NP67 and NP51) (Figure 2). Based on imipenem susceptibility testing, the CLP *Φ*4 was most effective in killing imipenem‐nonsusceptible *P. aeruginosa* (66.67%) (Figure 2).

**Figure 3:**
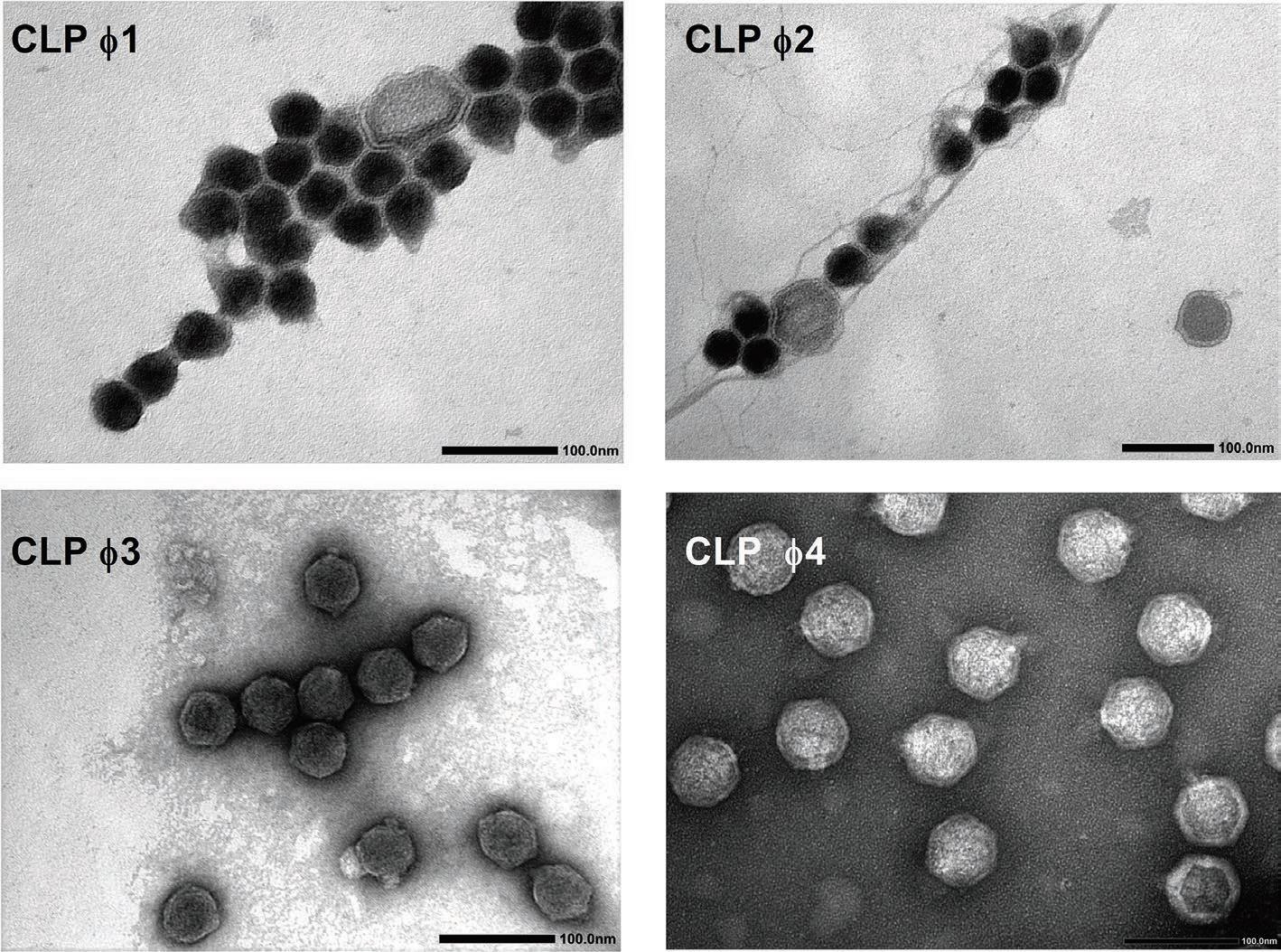
Morphology of CLP bacteriophages (*Φ*1–*Φ*4) observed under the transmission electron microscope (TEM).

In addition, the morphology of bacteriophages examined under the TEM showed that the CLP *Φ*1 to *Φ*4 were tailed phages belonging to the Caudoviricetes class, and had short noncontractile tails, indicating the Podoviridae (Figure 3A–D). In conclusion, the CLP mouse feces contained podoviruses that were efficacious against *P. aeruginosa*, including carbapenem‐resistant strains, suggesting the CLP *Φ*4 for further investigation.

### 3.3. Characterization of Replication Kinetics and Antibacterial Activity of the CLP *Φ*4

**Figure 4:**
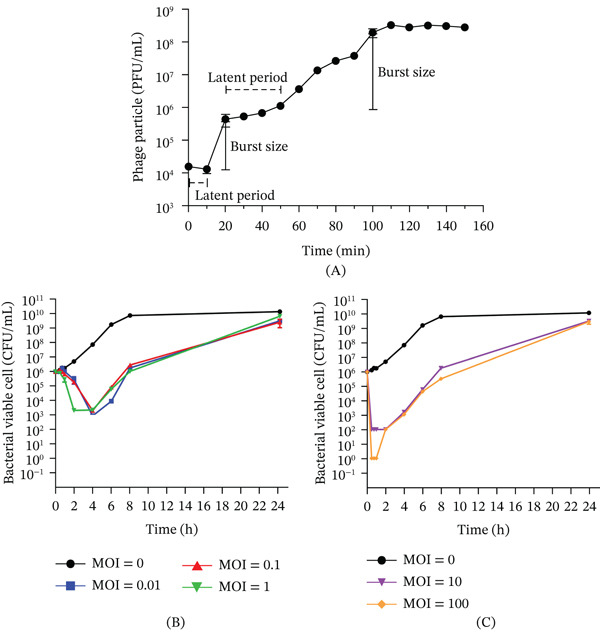
One‐step growth curve and time‐kill curve of the phage CLP *Φ*4. (A) The one‐step growth curve demonstrated the replication kinetics with burst sizes of the phage CLP *Φ*4. (B and C) The time‐kill curve demonstrated bactericidal activity of the phage CLP *Φ*4 against *P. aeruginosa* PACL. The experiments were performed in independent triplicate. All data were presented as median with range.

To characterize the in vitro activity of the CLP *Φ*4, *P. aeruginosa* PACL, which exhibits wound infectivity and pneumonia in wound infection and pneumonia mouse models [12, 25]. The replication kinetics of the CLP *Φ*4 revealed two burst events during bacteriophage growth (Figure 4A). According to the one‐step growth curve, the first burst size (33.08 PFU/infected cells) occurred after the first 10‐min latent period (Figure 4A). Subsequently, a latency of 30 min occurred, followed by a second burst (147.79 PFU/infected cells) (Figure 4). At the MOI = 0.01, 0.1, and 1 against *P. aeruginosa* PACL, the CLP *Φ*4 did not exhibit bactericidal activity (Figure 4B). In contrast, bactericidal activity was observed at the MOI of 10 and 100 using the time‐kill curve (Figure 4C). The MOI of 10 showed a killing effect for 2 h. Whereas the MOI of 100 exhibited antibacterial activity, resulting in undetectable bacteria and a killing effect for the second hour. These results demonstrated that the CLP *Φ*4 specifically killed *P. aeruginosa* by bactericidal activity. These results demonstrated that the CLP *Φ*4 specifically killed *P. aeruginosa* with bactericidal activity.

### 3.4. BMDMs Respond to the CLP *Φ*4 and *P. aeruginosa*

**Figure 5:**
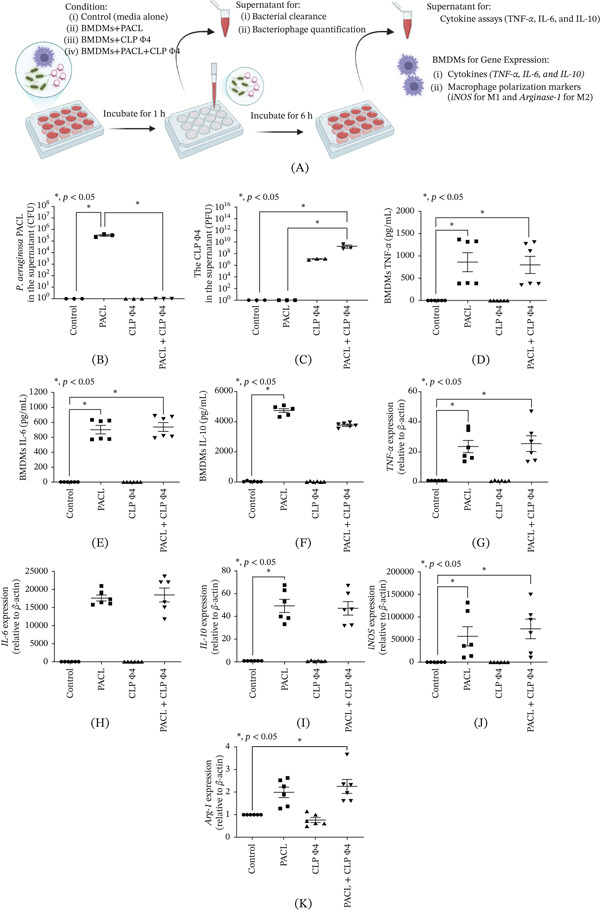
Diagram and cytokine response of bone marrow‐derived macrophages (BMDMs). (A) Either *P. aeruginosa* (*n* = 6), the CLP *Φ*4 (*n* = 6), or the combination (*n* = 6) was coincubated with BMDM. (B–C) The viable *P. aeruginosa* (*n* = 3) and CLP *Φ*4 (*n* = 3) in the supernatant were determined. (D, E, and F) The cytokines (TNF‐*α*, IL‐6, and IL‐10) in supernatant BMDM culture were determined. (G–K) The BMDMs were collected and evaluated for the expression of cytokine and macrophage‐polarizing genes (TNF‐*α*, *IL-6*, *IL-10*, *iNOS*, and *Arg-1*). All data were presented as median with range.

Before studying the CLP *Φ*4 in a mouse model, the immune response of BMDMs was studied using an ex vivo assay under conditions designed as shown in Figure 5A. The coculture of *P. aeruginosa* PACL and the CLP *Φ*4 resulted in complete bacterial clearance and elevated CLP *Φ*4 particles (Figure 5B,C). *P. aeruginosa* PACL promoted the secretion of proinflammatory cytokines (TNF‐*α* and IL‐6) and anti‐inflammatory cytokine (IL‐10), indicating the strongly induced BMDM response (Figure 5D–F). There was no BMDM response against the CLP *Φ*4 alone, indicating a slightly affected mammalian immune cell response. The PACL alone and the coculture of PACL + *Φ*4 showed similar production of cytokines by the BMDMs, suggesting no interference of bacteriophage against *P. aeruginosa* infection. In addition, the cytokine gene expression of the BMDMs showed concordant results with the secreted cytokines (Figure 5G–I). *TNF-α*, *IL-6*, and *IL-10* genes were overexpressed in PACL alone and the coculture of PACL + *Φ*4. Furthermore, the expression of macrophage polarization genes was upregulated by the PACL alone and the coculture of PACL + *Φ*4, including *iNOS* and *Arg-1*, confirming a simultaneous response to M1 (proinflammatory) and M2 (anti‐inflammatory), respectively (Figure 5J,K).

### 3.5. The CLP *Φ*4 Eradicates *P. aeruginosa* PACL in a Mouse Wound Model

**Figure 6:**
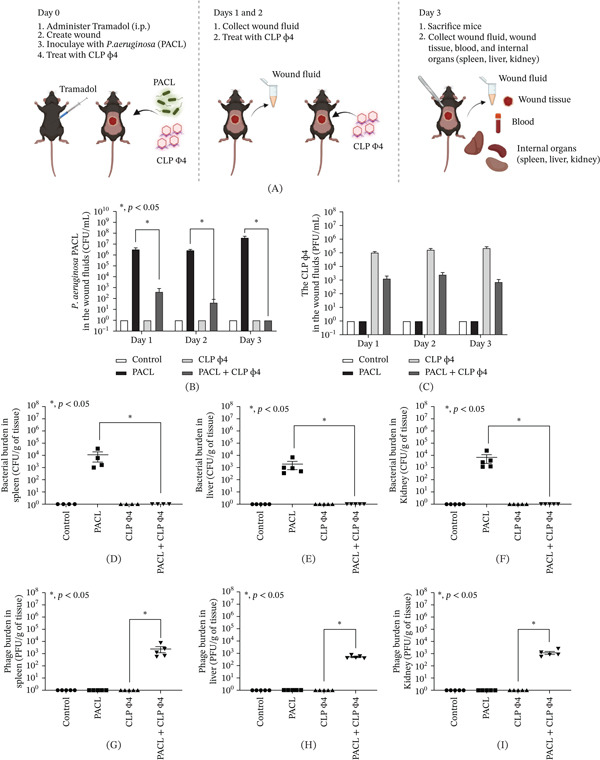
Diagram of the *P. aeruginosa*–infected wound model and bacterial and bacteriophage burdens study (*n* = 5/group). (A) The treatment of the *P. aeruginosa* PACL‐infected wound mouse model was using the CLP *Φ*4. The viable cells of *P. aeruginosa* PACL and the CLP *Φ*4 in daily collected (B–C) wound fluids and (D–I) internal organs, including spleen, liver, and kidney, were determined using plate counting and double‐layer agar methods. All data were present as median with range.

Accordingly, the CLP *Φ*4 revealed an in vitro bactericidal activity against *P. aeruginosa* PACL. Therefore, the curative effect of CLP *Φ*4 against *P. aeruginosa* was studied using a *P. aeruginosa*–infected mouse wound model under conditions designed as shown in Figure 6A. Daily collected wound fluids showed increased PACL burden in the PACL‐infected group. In contrast, the bacterial burden of the CLP *Φ*4 treatment group significantly decreased with the presence of the CLP *Φ*4, indicating the eradication efficacy of the CLP *Φ*4 in the wound model (Figure 6B,C). In the *P. aeruginosa* infection group, PACL was isolated from the spleen, liver, and kidney (Figure 6D–F). Interestingly, the CLP *Φ*4 treatment group showed no PACL burdens in any organs, indicating complete clearance (Figure 6D–F). For bacteriophage burdens, the CLP *Φ*4 was found in the spleen, liver, and kidney of the treatment group (Figure 6G–I). Therefore, the CLP *Φ*4 eradicated *P. aeruginosa* in the wounds and internal organs.

### 3.6. Impact of the CLP *Φ*4 on the Severity of *P. aeruginosa* PACL‐Infected Wounds

**Figure 7:**
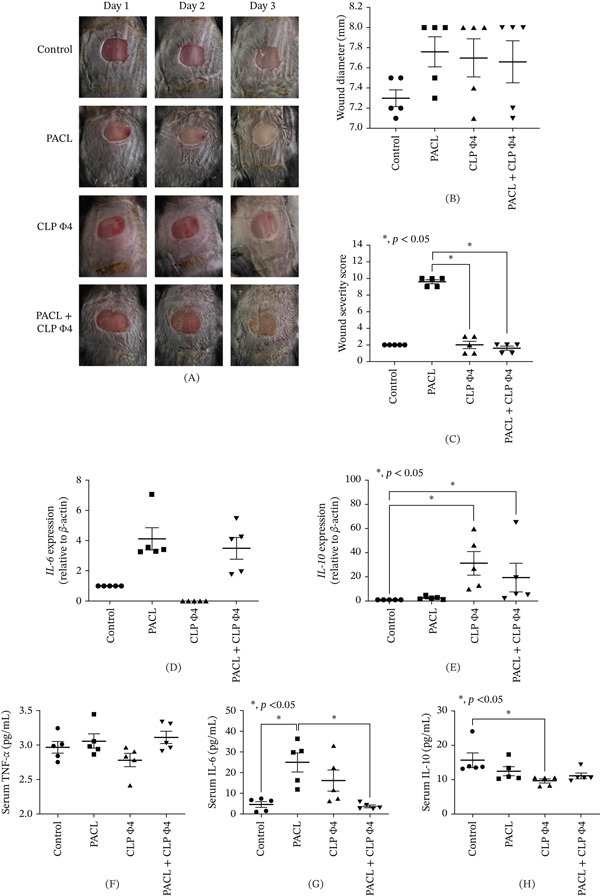
The severity of *P. aeruginosa*–infected wounds treatment with the CLP *Φ*4 (*n* = 5/group). (A) Wound pictures, (B) wound diameters, and (C) wound severity scores on the third day of the treatment are presented. (D–E) The wound tissue cytokine‐gene expressions determined by RT‐qPCR are shown. (F–H) The mouse serum cytokines evaluated by ELISA are shown. All data were presented as median with range.

The represented pictures of wounds in each group on Days 1, 2, and 3 in Figure 7A revealed that the CLP *Φ*4 treatment resulted in reduced inflammation. Although the wound diameters on Day 3 of wounds in each group were comparable (Figure 7B), the overall scores of wound severities (including wound diameter, infiltrating cells, redness, and moisture) in the CLP *Φ*4 treatment exhibited a remarkably decreased severity score (Figure 7C). Consistently, the wound tissues in the treatment group showed a reduced IL‐6 expression (Figure 7D) but elevated IL‐10 level (Figure 7E). Additionally, the systemic severity evaluated by mouse serum cytokines (Figure 7F–H) revealed that there were neither different levels of TNF‐ *α* nor IL‐10 but a notably decreased IL‐6 cytokine in the treatment group.

### 3.7. The CLP *Φ*4 Treatment Decreases Infiltrating Cells in *P. aeruginosa* PACL‐Infected Wounds

**Figure 8:**
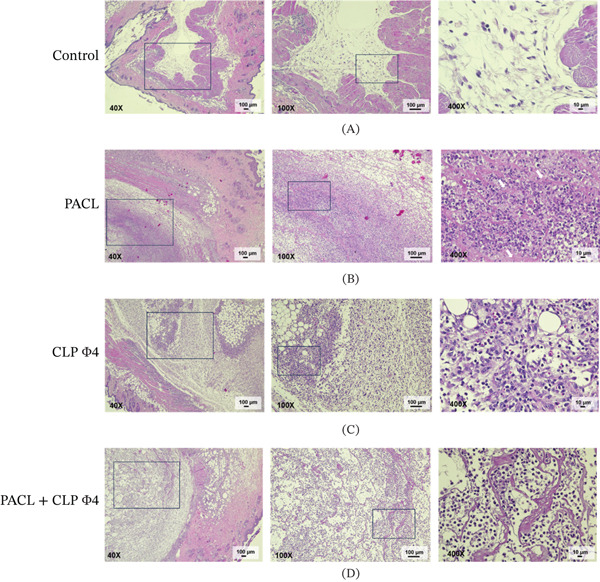
Histopathological examination of mouse wound tissues (*n* = 5/group). The representative images of microscopically examined wound tissues from the (A) control, (B) *P. aeruginosa* PACL infection, (C) the CLP *Φ*4 alone, and (D) the *P. aeruginosa* PACL infection with the CLP *Φ*4 treatment.

Accordingly, there were notable macroscopic differences in the wounds (Figure 7A). The mouse wound tissues stained with H&E were studied using a light microscope. Wound‐noninfected control tissues showed few infiltrating cells (Figure 8A), whereas *P. aeruginosa* PACL‐infected wounds had a tremendous number of cells (Figure 8B). In the CLP *Φ*4 alone and the treatment groups, the tissues showed decreased cell infiltration (Figure 8C,D).

## 4. Discussion

The World Health Organization (WHO) announced a list of priority pathogens, including *Acinetobacter*, *Pseudomonas,* and various Enterobacteriaceae, as the most critical group, requiring strict guidelines and new antibiotic development [27]. Many studies have previously focused on antibiotic combinations [28–30]; however, rapidly emerging resistant bacteria and the discontinuation of novel antibiotics are important obstacles. Another strategy is bacteriophage therapy, which was first used in 1915 but is raising interest today [31]. We previously isolated effective *P. aeruginosa* phages from animal sources (wild‐type mice) for a pneumonia therapeutic model [12]. In this study, we successfully isolated CLP bacteriophages specific to *P. aeruginosa* from inflammatory mice (CLP model), answering our primary objective based on the presence of podovirus predominance in the CLP mouse feces [15]. Gut dysbiosis by dominant Caudoviricetes was associated with inflammatory bowel disease (IBD), possibly due to the killing of enteric normal flora [32]. In contrast, the sepsis animal with gut dysbiosis contained *P. aeruginosa* podovirus, indicating that not every Caudoviricetes is a villain [15]. The CLP mouse feces richness of Caudoviricete, especially Podoviridae, can attenuate sepsis severity in mouse models, suggesting the beneficial bacteriophages in the CLP feces [15].

The CLP *Φ*4 exhibited the broadest spectrum with two large burst sizes related to bactericidal activity against *P. aeruginosa*, suggesting a promising therapeutic. Although there was an antimicrobial effect, the CLP *Φ*4 had no impact on BMDM immune response in a coculture with *P. aeruginosa*, suggesting nonimmunogenicity. However, various phages induced anti‐inflammatory responses, suggesting a strain‐dependent manner [33]. *P. aeruginosa* PACL was isolated from wounds (indicating localized infection), lymphatic organs (spleen and liver) (indicating migration to the immune system), and kidneys (indicating possibly early stages of systemic infections). The CLP *Φ*4 (topical use) completely eradicated *P. aeruginosa* in the infected wound within 3 days. Additionally, the CLP *Φ*4 in the mouse internal organs eliminated *P. aeruginosa* in these organs, demonstrating that the bacteriophage traveled into the body along site with the *P. aeruginosa* host. Likewise, *P. aeruginosa* and bacteriophages were isolated from serum and bronchoalveolar lavage fluids in both intravenous and intranasal routes of pneumonia mice with septicemia in our previous study [12]. Furthermore, there was no CLP *Φ*4 in the organs of the CLP *Φ*4 alone, possibly due to a lack of host cells for replication. Additionally, the wide range of phage topical dose (10^7^–10^9^ PFU/mL) for wound treatment has been effective in clinical wound treatment, so our phage with a dose of 10^8^ PFU/mL was feasible for clinical application [34].

The healing process of the open wound is composed of hemostasis, inflammation, proliferation, and remodeling, respectively [35]. Hemostasis is the first step to stop bleeding, followed by the inflammation stage in 2–3 days. The proliferation phase of granulation tissue is a newly formed tissue composed of inflammatory cells, fibroblasts, and endothelial cells to connect the edge of the open wound after 7 days, followed by wound remodeling [35, 36]. Therefore, our 3‐day wound model exhibited the inflammatory stage. In the control group, the macroscopic wounds showed no filling of the wound gap, and there were rare infiltrating cells in the microscopic detection, indicating the inflammatory stage [25]. Interestingly, in the CLP *Φ*4 alone and treatment groups, more cell migration and filling of wound gaps were observed compared with the control group. Bacteriophages act as immunomodulators, balancing inflammatory and anti‐inflammatory cytokines [37]. Likewise, in the *P. aeruginosa*–infected group, not only bacterial eradication but also wound gap appeared after phage therapy, confirming the antibacterial and immune‐modulating activities of phages [35]. IL‐6, a proinflammatory cytokine, is responsible for inflammation and infection, whereas IL‐10, an anti‐inflammatory cytokine, counteracts inflammation [38, 39]. The most severe was the infection group, which showed numerous immune cells, indicating the difficulty of bacterial clearance [40, 41]. In contrast, bacteriophage treatment was effective in eliminating bacteria and immune balancing, confirming the dual efficacy of phages on infected wound therapy [42]. Moreover, in the presence of only bone marrow‐derived macrophages (BMDMs), the CLP *Φ*4 had no immune response effect but showed an immunomodulating effect in the mouse wound tissue, indicating the comprehensive property of bacteriophage, in which other immune or stromal cells involved—such as neutrophils, macrophages, or mesenchymal cells—might respond to phage exposure. Further investigation of these cellular interactions could provide a better understanding of phage–host dynamics, resulting in not only bacterial clearance but also immunomodulation.

The limitations of our study included (1) no bacteriophage genome study because our hypothesis was inflammation feces as a source of pathogen virulence phages, not finding a novel phage; however, the nucleotide sequence should be further analyzed before clinical use; (2) no antibiotic treatment control in this study to avoid the interference of the immune response by antibiotic; however, the standard wound dressing with daily change of covered film applied in all groups; and (3) no other parameter of wound healing study. The healing process of noninfected wounds usually occurs within 3–5 days [35, 40]. However, the *P. aeruginosa*–infected wound model exhibited systemic infection involvement that might interfere with local phage therapy, which was our objective [25]. The mice had signs of early systemic complications (the presence of bacteria in the kidneys) after 3 days of infection; therefore, a 3‐day treatment performed in this study might be inappropriate for a wound repair study. Thus, our experiment was a short‐term proof‐of‐concept of antibacterial activity, not wound healing property. Further studies are fascinating for CLP *Φ*4 and wound healing.

## 5. Conclusions

The inflammatory gut acted as a promising source of *P. aeruginosa* virulence bacteriophages that exhibited a broad host range and ultimate bactericidal activity, thereby proving the concept that the inflammatory gut microbiome might be a source of therapeutic phages. The bacteriophage eradicated *P. aeruginosa* burdens in infected open wounds and simultaneously counterbalanced inflammatory and anti‐inflammatory responses.

**Nomenclature**
BMDM: Bone marrow‐derived macrophage
CLP: Cecal ligation and puncture
DTR: Difficult‐to‐treat resistance
ECM: Extracellular matrix
NET: Neutrophil extracellular trap
PACL: *Pseudomonas aeruginosa* clinical isolate

## Author Contributions

U.S‐I. and A.L. conceptualized the experiments and methodology and supervised the project. U.S‐I. and S.P. investigated, collected, and analyzed the bacteriophage isolation, antibacterial activity, and ex vivo BMDM results. V.S. performed virome analysis. W.S. and P.P. isolated BMDM and investigated the mouse wound model. U.S‐I. investigated bacterial and bacteriophage burdens, cytokine production, gene expression, and the histological examination, and was a major contributor in writing the manuscript. U.S‐I., S.P., and A.L. wrote the original draft manuscript.

## Funding

This study was supported by the Rachadapiseksompotch Fund, Faculty of Medicine, Chulalongkorn University, Grant Number RA66/009.

## Disclosure

The funding institution has no role in the design of this study, work execution, analysis, interpretation of the data, and manuscript writing or submission. All authors read and approved the final manuscript.

## Ethics Statement

This study was ethically approved by the Institutional Animal Care and Use Committee of the Faculty of Medicine, Chulalongkorn University (Protocol Number 2591001) and the institutional review board (IRB), Faculty of Medicine, Chulalongkorn University (IRB number MDCU‐IBC012/2025).

## Consent

The authors have nothing to report.

## Conflicts of Interest

The authors declare no conflicts of interest.

## Supporting information


**Supporting Information** Additional supporting information can be found online in the Supporting Information section. Table S1: Primer used in this study. Table S2: Wound severity scoring criteria. Figure S1: RAPD profiles of *Pseudomonas aeruginosa* strains ATCC 27853, PAO1, PACL, PA1, PA2, PA4, PA5, PA6, PA7, PA9, PA10, PA11, PA12, PA13, and PA14. Figure S2: RAPD profiles of *Pseudomonas aeruginosa* strains NP8, NP37, NP62, NP98, NP100, NP101, NP107, NP10, NP46, NP54, NP55, NP60, NP18, NP23, and NP39. Figure S3: RAPD profiles of *Pseudomonas aeruginosa* strains NP51, NP52, NP53, NP56, NP57, NP67, NP82, NP85, NP95, and NP102.

## Data Availability

The data that supports the findings of this study are available in the Supporting Information of this article.
