# Supplementary material for: Pseudomonas aeruginosa Virulence Bacteriophage Isolated From Inflammatory Mouse Feces Exhibits Bactericidal Activity in Infected Wounds of a Mouse Model
Source: Int J Microbiol. 2026 Jul 3;2026:4451708. doi: 10.1155/ijm/4451708 (PMC13329690; doi:10.1155/ijm/4451708)
Supplement: Supplementary file 1 — Supporting Information Additional supporting information can be found online in the Supporting Information section. Table S1: Primer used in this study. Table S2: Wound severity scoring criteria. Figure S1: RAPD profiles of Pseudomonas aeruginosa strains ATCC 27853, PAO1, PACL, PA1, PA2, PA4, PA5, PA6, PA7, PA9, PA10, PA11, PA12, PA13, and PA14. Figure S2: RAPD profiles of Pseudomonas aeruginosa strains NP8, NP37, NP62, NP98, NP100, NP101, NP107, NP10, NP46, NP54, NP55, NP60, NP18, NP23, and NP39. Figure S3: RAPD profiles of Pseudomonas aeruginosa strains NP51, NP52, NP53, NP56, NP57, NP67, NP82, NP85, NP95, and NP102. [file IJM-2026-4451708-s001.docx]

**Supplementary Table 1. Primer used in this study.**

| **Primer name** | **Gene name** | **Oligonucleotide sequence (5ˊ-3ˊ)** |
| --- | --- | --- |
| 10514 | RAPD typing | TGGTGGCCTCGAGCAAGAGAACGG |
| TNF-α-F | *TNF-α* | CCTCACACTCAGATCATCTTCTC |
| TNF-α-R |  | AGATCCATGCCGTTGGCCAG |
| IL-6-F | *IL-6* | ATGAACTCCTTCTCCACAAGC |
| IL-6-R |  | GTTTTCTGCCAGTGCCTCTTTG |
| IL-10-F | *IL-10* | TCTCCGAGATGCCTTCAGCAGA |
| IL-10-R |  | TCAGACAAGGCTTGGCAACCCA |
| iNOS-F | *iNOS* | ACCCACATCTGGCAGAATGAG |
| iNOS-R |  | AGCCATGACCTTTCGCATTAG |
| Arg-1-F | *Arg-1* | CTTGGCTTGCTTCGGAACTC |
| Arg-r-R |  | GGAGAAGGCGTTTGCTTAGTTC |
| β-actin-F | *β-actin* | CCTGGCACCCAGCACAAT |
| β-actin-R |  | GCCGATCCACACGGAGTACT |

**Supplementary Table 2. Wound severity scoring criteria**

| **Score** | **0** | **1** | **2** | **3** |
| --- | --- | --- | --- | --- |
| 1. Wound diameter (mm) | <8 mm | 8 mm | 9 mm | >9 mm |
| 2. Infiltrated cell | No | Few | Moderate | Numerous |
| 3. Redness | No redness | Mild, present | Moderate | Severe |
| 4. Moisture | Dry | Mild, present | Moderate | Severe |


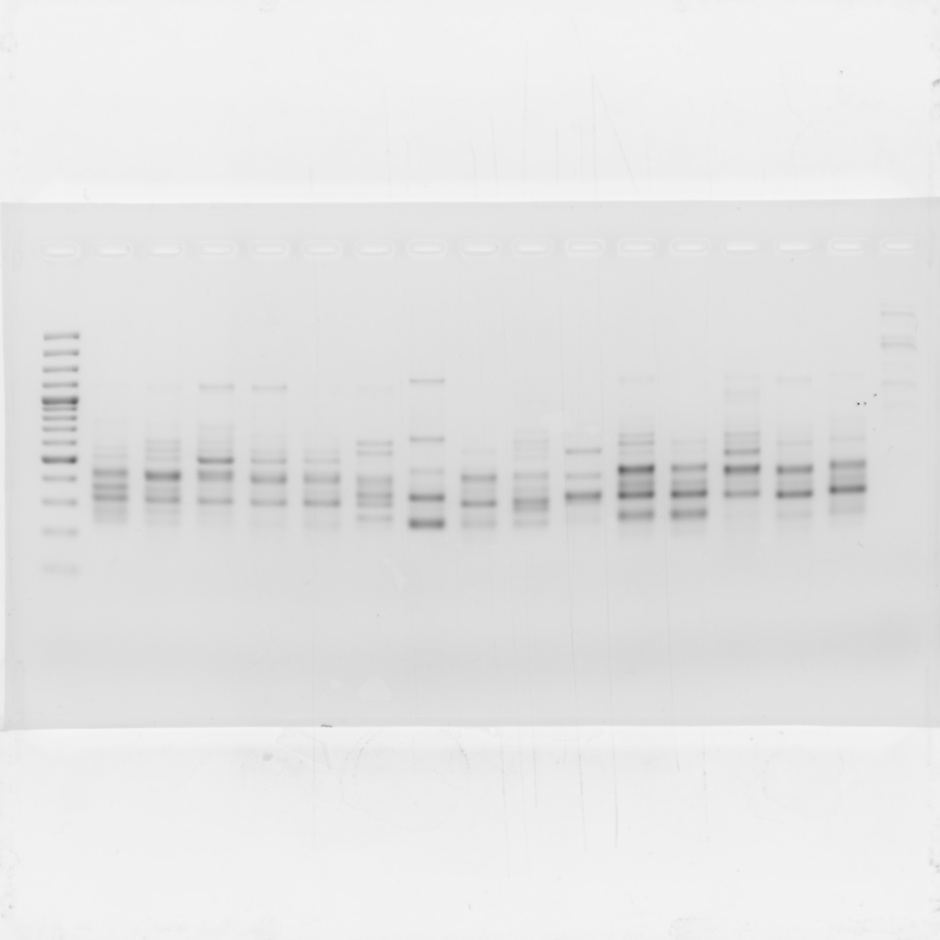


**Supplementary Figure 1.** RAPD profiles of *Pseudomonas aeruginosa* strains ATCC 27853, PAO1, PACL, PA1, PA2, PA4, PA5, PA6, PA7, PA9, PA10, PA11, PA12, PA13, and PA14.


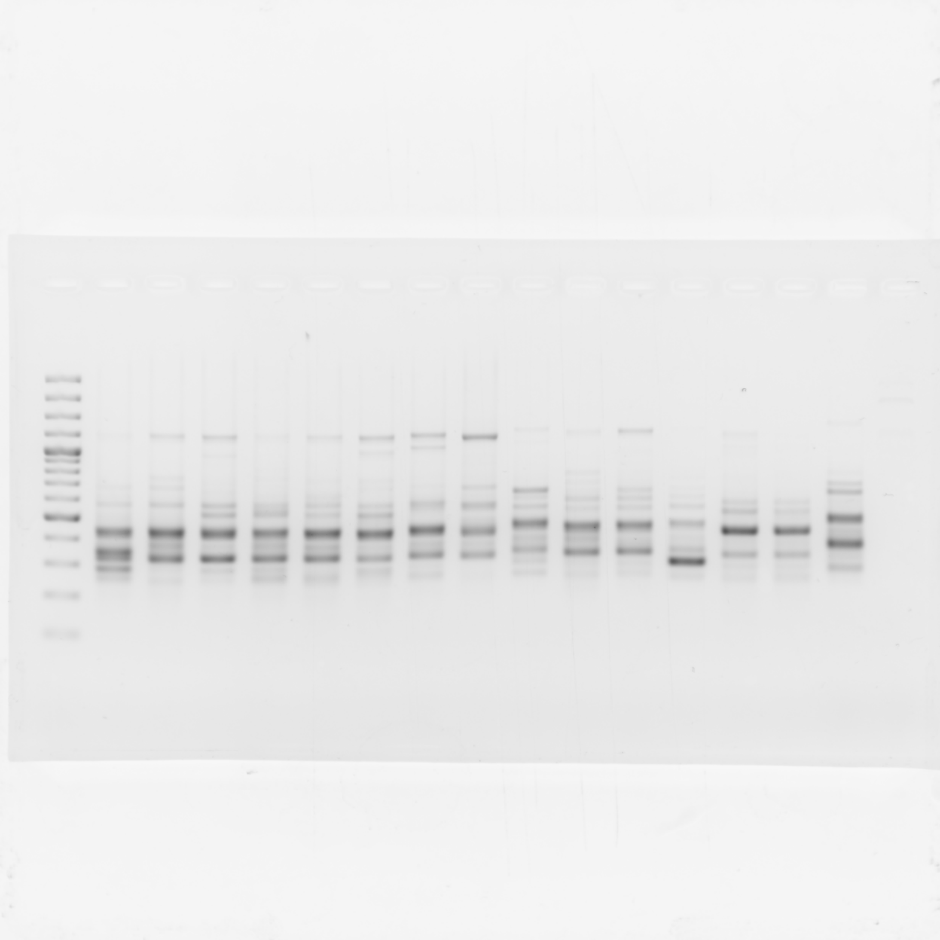


**Supplementary Figure 2.** RAPD profiles of *Pseudomonas aeruginosa* strains NP8, NP37, NP62, NP98, NP100, NP101, NP107, NP10, NP46, NP54, NP55, NP60, NP18, NP23, and NP39.


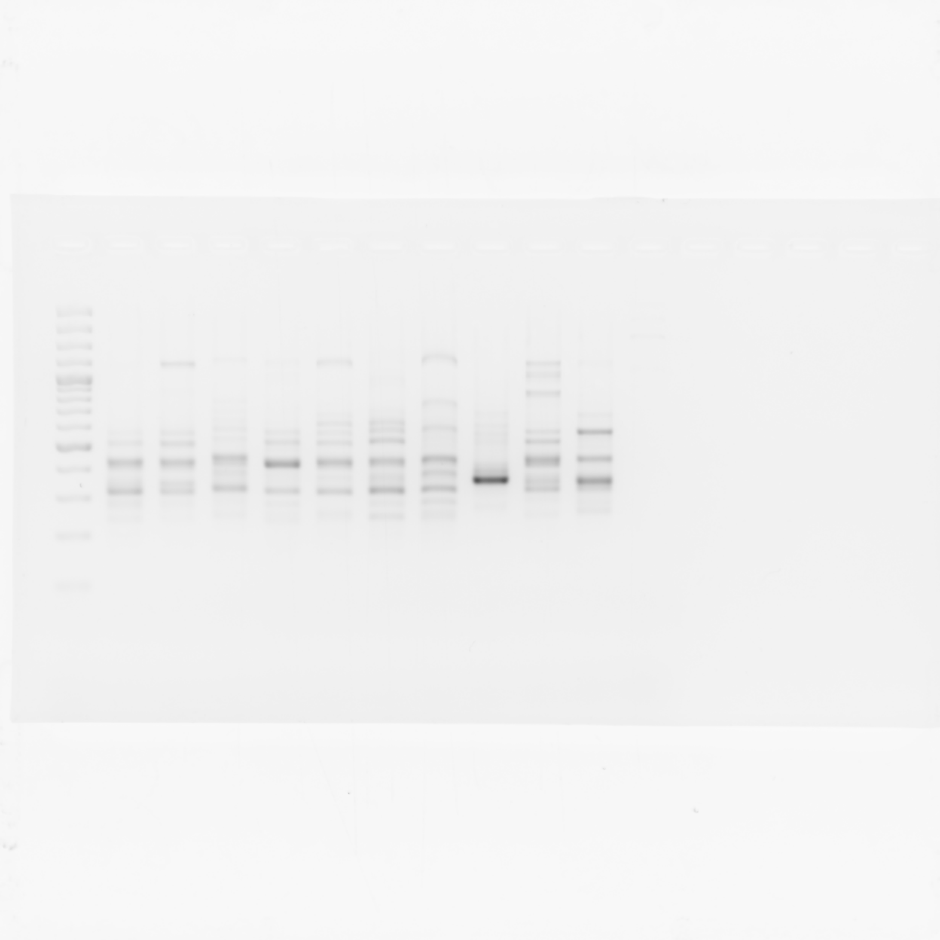


**Supplementary Figure 3.** RAPD profiles of *Pseudomonas aeruginosa* strains NP51, NP52, NP53, NP56, NP57, NP67, NP82, NP85, NP95, and NP102.
